# Factors associated with extremely poor visual outcomes in patients with central retinal vein occlusion

**DOI:** 10.1038/s41598-020-76840-6

**Published:** 2020-11-12

**Authors:** Daisuke Nagasato, Yuki Muraoka, Rie Osaka, Yuko Iida-Miwa, Yoshinori Mitamura, Hitoshi Tabuchi, Shin Kadomoto, Tomoaki Murakami, Sotaro Ooto, Kiyoshi Suzuma, Akitaka Tsujikawa

**Affiliations:** 1 Department of Ophthalmology, Saneikai Tsukazaki Hospital, Himeji, Hyogo Japan; 2 Department of Ophthalmology, Institute of Biomedical Sciences, Tokushima University Graduate School, Tokushima, Japan; 3 Department of Ophthalmology and Visual Sciences, Kyoto University Graduate School of Medicine, 54 Shougoin Kawahara-cho, Sakyo-ku, Kyoto, 606-8507 Japan; 4 Department of Ophthalmology, Kagawa University Faculty of Medicine, Kagawa, Japan

**Keywords:** Diseases, Medical research

## Abstract

Here, we examined prognostic factors for extremely poor visual outcomes in patients with central retinal vein occlusion (CRVO) in actual practices. We included 150 consecutive eyes with treatment-naïve acute CRVO from four different facilities and observed them for over 24 months. Macular edema (ME) was treated with one or three monthly anti-vascular endothelial growth factor injections (1 or 3 + pro re nata). According to the final Snellen visual acuity (VA), we divided the patients into very poor VA (< 20/200) and control (≥ 20/200) groups and examined risk factors for poor final visual outcomes. The baseline Snellen VA was hand motion to 20/13. The mean number of anti-VEGF injections for ME was 5.3 ± 3.7 during the follow-up period. In total, 49 (32.7%) patients exhibited a very poor final VA; this group comprised significantly older patients with a significantly poorer baseline VA (*P* < 0.01 for both) than the control group. Comorbid internal carotid artery disease and diabetic retinopathy were significantly associated with a poor final VA. In actual clinical practice, visual outcomes may be extremely poor despite ME treatment in certain patients with CRVO, with advanced age, poor baseline VA, and comorbid internal carotid artery disease and diabetic retinopathy being significant risk factors.

## Introduction

Central retinal vein occlusion (CRVO) is a common retinal vascular disorder. Because of circulatory disturbances in the trunk of the central retinal vein, the retinal nonperfusion area (NPA) can extend to the entire retina^1^. Thus far, the status of the retinal circulation in eyes with CRVO has been classified according to the definition in the Central Vein Occlusion Study^2–6^. Neovascular complications such as rubeosis and neovascular glaucoma (NVG) are more frequent in eyes with ischemic CRVO than in eyes with nonischemic CRVO; this is because of greater upregulation of vascular endothelial growth factor (VEGF) in the former. Considering the risk of these neovascular complications, clinical practice for eyes with CRVO may be considerably different from that for eyes with branch retinal vein occlusion or hemi-CRVO^1,7,8^.

Recent randomized clinical trials have reported good visual outcomes after anti-VEGF treatments for macular edema (ME) in eyes with both nonischemic and ischemic CRVO^9–15^, suppression of the retinal exudate or possible inhibition of NPA enlargement might have played a role in these visual outcomes^16^. However, the status of the retinal circulation in the eyes included in these clinical trials was probably not very poor, because the incidence of neovascular complications was lower than that observed in the natural course of ischemic CRVO^13^. Other clinical trials including patients with severe CRVO reported that VEGF blockade by anti-VEGF treatments did not lower the risk of neovascular complications^17^. Therefore, we consider that the visual outcomes of eyes with CRVO, including severe cases, that are encountered in actual clinical practice may be poorly evaluated.

Accordingly, the aim of this multicenter study was to evaluate visual outcomes and examine the factors associated with extremely poor visual outcomes in patients with CRVO.

## Results

In total, 150 eyes of 150 consecutive patients (mean age: 69.2 ± 12.8 years; 97 men and 53 women) with unilateral, treatment-naïve, acute CRVO were included. At the initial visit, all eyes (100%) showed retinal hemorrhage and dilated tortuous veins throughout the retina and ME and/or serous retinal detachment at the fovea. Table 1 shows the systemic and ocular manifestations of the included patients. The mean initial logMAR BCVA was 0.67 ± 0.52 (Snellen equivalent: hand motion to 20/13), and the mean number of anti-VEGF injections for ME was 5.3 ± 3.7 during the follow-up period of 34.9 ± 16.1 months. Seventy-seven eyes in Tsukazaki Hospital and Tokushima University Hospital initially received three monthly injections of aflibercept or ranibizumab, while 44 eyes in Kyoto University Hospital initially received three monthly aflibercept injections. In Kagawa University Hospital, 29 eyes received one initial aflibercept or ranibizumab injection.

**Table 1:** Characteristics of included 150 patients with central retinal vein occlusion.

Systemic hypertension (n, %)	95 (63.3)
Hyperlipidemia (n, %)	33 (22.0)
Diabetes (n, %)	38 (25.3)
Smoking history (n, %)	36 (24.0)
Internal carotid artery disease (n, %)	6 (4.0)
Coronary artery disease (n, %)	13 (8.7)
Dialysis (n, %)	2 (1.3)
Diabetic retinopathy (n, %)	15 (10.0)
Glaucoma (n, %)	15 (10.0)
Number of intravitreal anti-VEGF injections	5.3 ± 3.7

Data are expressed as mean ± standard deviation unless otherwise indicated.

Patients with diabetes were included if they showed no diabetic retinopathy or mild to moderate nonproliferative diabetic retinopathy without macular edema.

**Figure 1:**
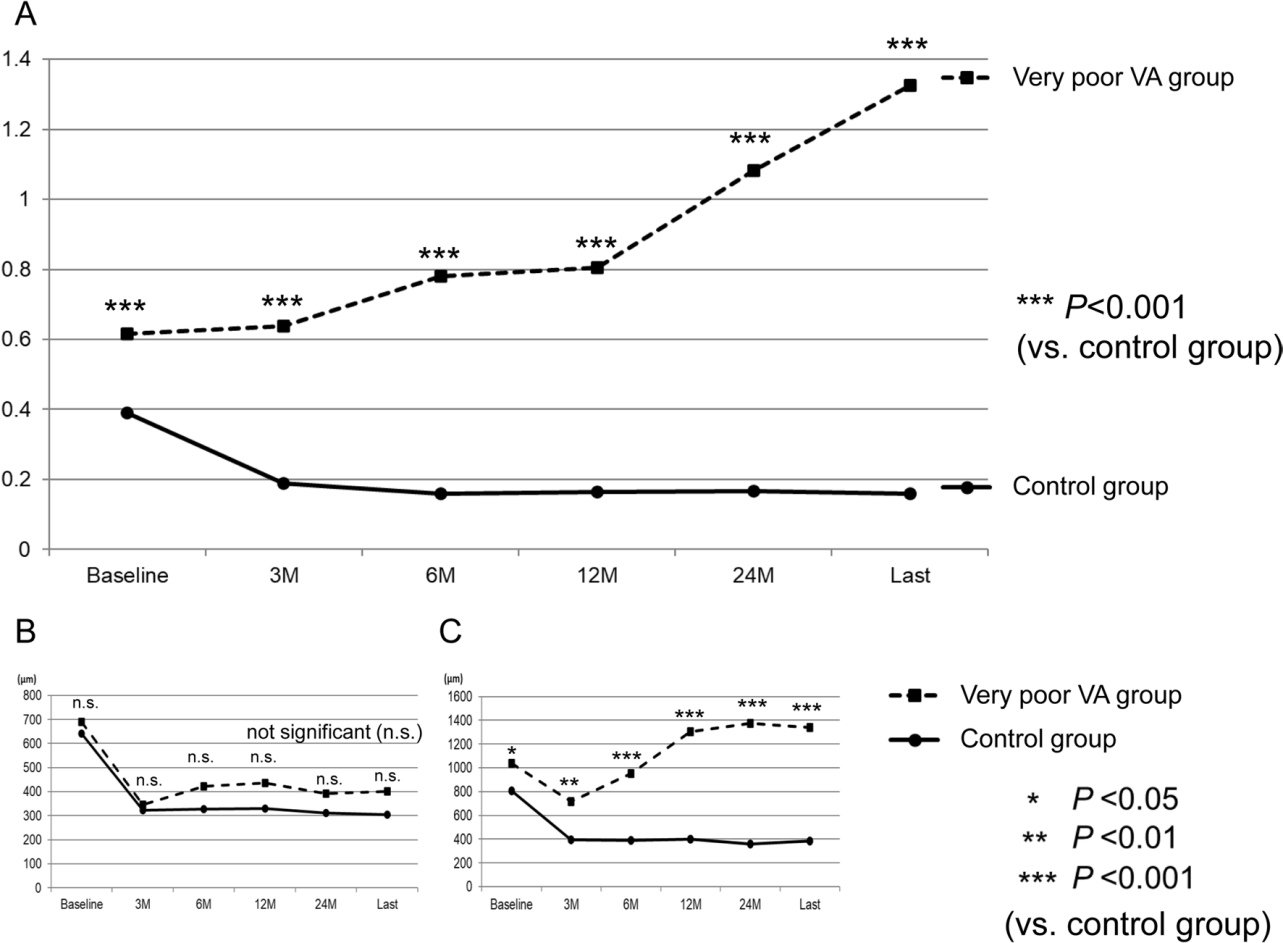
Comparisons of the best-corrected visual acuity (logarithm of the minimum angle of resolution) (**A**), central retinal thickness (**B**), and defect length in the ellipsoid zone band (**C**) at corresponding time points between central retinal vein occlusion-patients exhibiting a final Snellen visual acuity (VA) of < 20/200 (very poor VA group) or ≥ 20/200 (control group).

According to the final Snellen BCVA, we divided the patients into very poor VA (< 20/200) and control (≥ 20/200) groups. One-hundred and one patients (67.3%; 63 men and 38 women) were in the control group, and the final mean logMAR BCVA was 1.31 ± 0.57. Forty-nine patients (32.7%; 34 men and 15 women) were in the very poor VA group, and the final mean logMAR BCVA was 0.24 ± 0.33. The sex distribution, duration from onset to the initial visit, mean number of anti-VEGF injections, and initial and final CRTs were not significantly different between the two groups (Table 2, Fig. 1). The baseline BCVA was significantly poorer while the patients were significantly older in the very poor VA group than in the control group (*P* < 0.01 for both; Table 2, Fig. 1).

**Table 2:** Characteristics of included patients with central retinal vein occlusion stratified according to the final snellen visual acuity (≥ 20/200 and < 20/200).

	All (n = 150)	Control group (n = 101)	Very poor VA group (n = 49)	*P* value
Sex (men/women)	97/53	63/38	34/15	0.399
Age (years)	69.2 ± 12.8	67.2 ± 13.3	73.4 ± 10.4	0.002
Duration of symptoms (weeks)	6.8 ± 11.8	5.4 ± 7.2	8.8 ± 17.5	0.193
Observation period (months)	34.9 ± 16.1	32.9 ± 16.9	35.4 ± 18.3	0.467
Baseline logMAR BCVA (Snellen equivalent)	0.67 ± 0.54 (HM to 20/13)	0.54 ± 0.45 (20/2000 to 20/13)	0.93 ± 0.61 (HM to 20/25)	< 0.001 NA
Baseline CRT (µm)	656 ± 255	641 ± 229	691 ± 314	0.287
Final logMAR BCVA (Snellen equivalent)	0.60 ± 0.67 (NLP to 20/13)	0.24 ± 0.33 (20/200 to 20/13)	1.31 ± 0.57 (NLP to 20/228)	< 0.001 NA
Final CRT (µm)	329.3 ± 171.8	304.9 ± 149.4	338.2 ± 231.9	0.367
Number of anti-VEGF injections	5.3 ± 3.7	5.4 ± 3.4	5.2 ± 4.2	0.765

Data are expressed as mean ± standard deviation unless otherwise indicated.

*BCVA* best-corrected visual acuity, *logMAR* logarithm of the minimum angle of resolution, *HM* hand motion, *NLP* no light perception, *NA* not applicable, *CRT* central retinal thickness, *VEGF* vascular endothelial growth factor.

**Figure 2:**
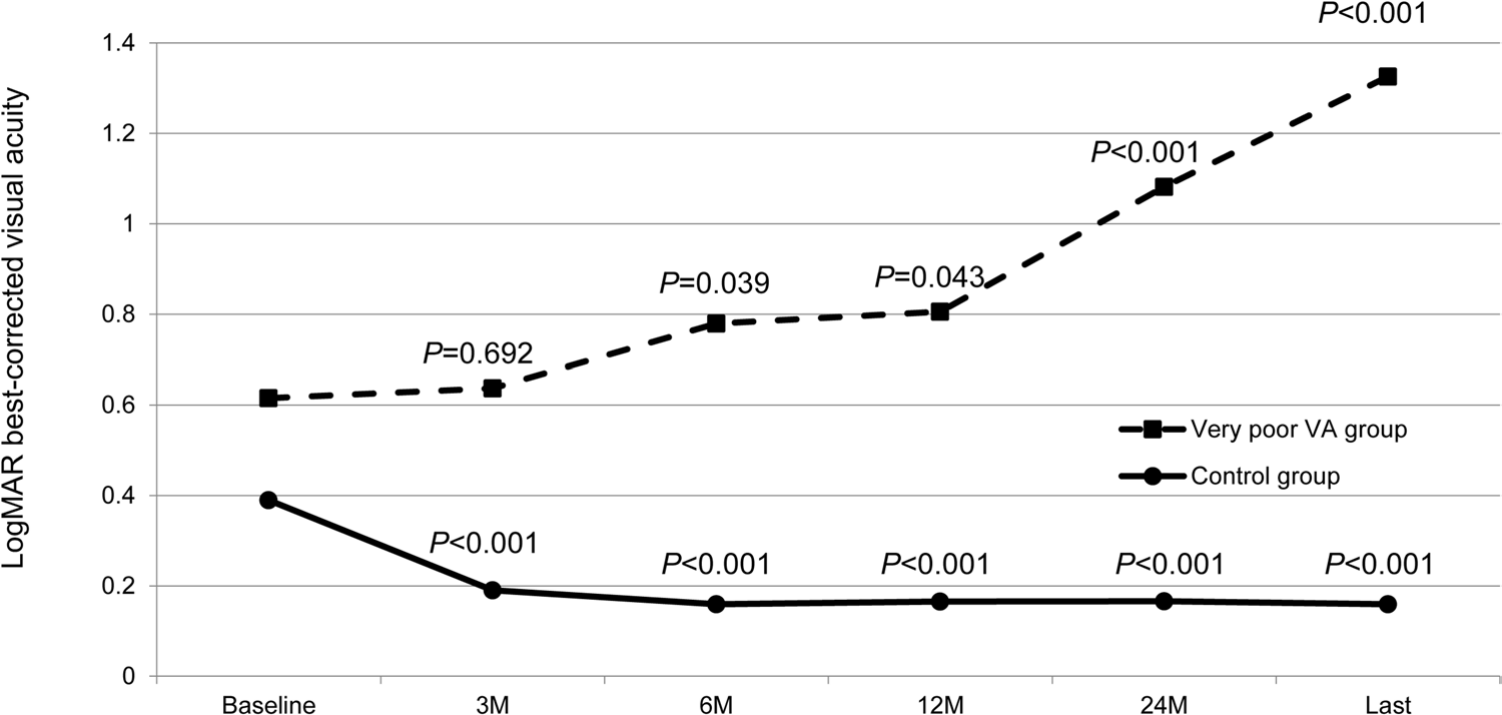
Longitudinal changes in best-corrected visual acuity (logarithm of the minimum angle of resolution) from baseline in patients with central retinal vein occlusion, exhibiting a final Snellen visual acuity (VA) of < 20/200 (very poor VA group) or ≥ 20/200 (control group). In the very poor VA group, the BCVAs gradually deteriorated. The BCVAs after month 3 are significantly worse than the baseline BCVA. In the control group, the BCVAs are significantly better compared to that at baseline (*P* < 0.001 for all measuring points).

**Figure 3:**
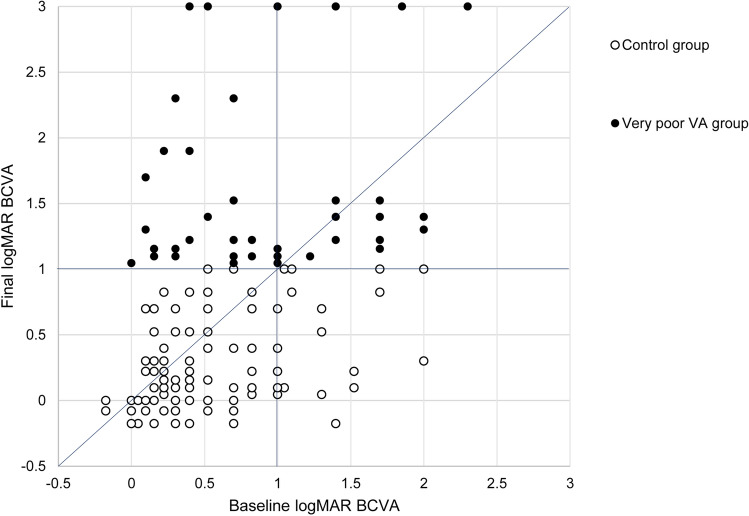
A scatter plot showing the best-corrected visual acuity (logarithm of the minimum angle of resolution) at baseline and final examination of each patient.

Figure 1 shows the longitudinal changes in the logMAR BCVA, CRT, and defect length in the EZ band for each group. In the control group, BCVA at 6, 12, and 24 months showed an improvement relative to the baseline BCVA (*P* < 0.001 for all). In the very poor VA group, BCVA showed a slight improvement at month 3 (the difference was not statistically significant); however, this improvement was not maintained, and BCVA at 24 months was significantly worse than the baseline BCVA (*P* < 0.001). Throughout the observational period, BCVA in the very poor VA group was significantly worse than that in the control group (*P* < 0.001 for all). Figure 2 shows longitudinal change in logMAR BCVA for each group. In the very poor VA group, the BCVAs gradually deteriorated. The BCVA at month 3 was not different from the baseline BCVA; however, the BCVAs after month 3 were significantly worse compared to that at baseline. In the control group, the BCVAs were significantly better after initiation of the anti-VEGF therapy compared to that at baseline (*P* < 0.001 for all measuring points). Figure 3 is a scatter plot showing the logMAR BCVA at baseline and final examinations for each patient.

In both groups, CRT at 6, 12, and 24 months was significantly lower than that at baseline (*P* < 0.001 for all) in the both two groups, with no significant between-group difference at any time point. The defect length in the EZ band was significantly greater in the very poor VA group than in the control group throughout the observational period (*P* < 0.001 for all).

In total, 22 (14.7%) eyes developed neovascular complications during the observational period; these included six eyes with rubeosis without IOP elevation and 16 eyes with NVG in the very poor VA group (Table 3). Vitreous hemorrhage occurred in 10 eyes, one of which belonged to the control group (Table 3). The mean logMAR BCVA for eyes with NVG was 0.70 ± 0.60 (Snellen BCVA, hand motion to 20/25) at baseline and 1.50 ± 0.92 (Snellen BCVA, no light perception to 20/250) at the final examination. In the very poor VA group, the final BCVA was worse than counting fingers for six eyes, all of which had developed NVG.

**Table 3:** Neovascular complications, additional treatments associated with a very poor final visual acuity (< 20/200) in patients with central retinal vein occlusion.

	Control group (n = 101)	Very poor group (n = 49)	*P* value
Vitreous hemorrhage (n; %)	1 (1.0)	9 (18.4)	< 0.001
Rubeosis without IOP elevation (n; %)	0 (0.0)	6 (12.2)	< 0.001
NVG (n; %)	0 (0.0)	16 (32.7)	< 0.001
Panretinal photocoagulation (n; %)	14 (13.9)	32 (65.3)	< 0.001
Glaucoma surgery for NVG (n; %)	0 (0.0)	5 (10.2)	0.001

Data are expressed as mean ± standard deviation unless otherwise indicated. IOP, intraocular pressure; NVG, neovascular glaucoma.

A final Snellen BCVA of < 20/200 was significantly associated with poor baseline BCVA, comorbid internal carotid artery disease, and comorbid diabetic retinopathy, with adjusted odds ratios (OR) of 5.96 (95% confidence interval [CI]: 1.88–18.86), 0.031 (95% CI: 0.78–24.91), and 0.05 (95% CI: 0.01–0.42), respectively (Table 4).

**Table 4:** Summary of the multivariate analyses for very poor final visual acuity (< 20/200) in patients with central retinal vein occlusion.

	Multivariate analyses			
	Beta	Standard error	Adjusted odds ratio (95%CI)	*P* value
Systemic hypertension	− 0.060	0.718	0.94 (0.23–3.85)	0.933
Hyperlipidemia	− 0.399	0.669	0.67 (0.18–2.49)	0.551
Smoking	0.288	0.702	1.33 (0.00–0.94)	0.682
Internal carotid artery disease	− 3.475	1.742	0.03 (0.78–24.91)	0.046
Coronary artery diseases	− 1.882	1.593	0.15 (0.01–3.46)	0.238
Dialysis	1.339	1.274	3.82 (0.31–46.36)	0.293
Diabetic retinopathy	− 3.071	1.121	0.05 (0.01–0.42)	0.006
Glaucoma	− 1.401	0.997	0.25 (0.04–1.74)	0.160
Baseline logMAR BCVA	1.785	0.588	5.96 (1.88–18.86)	0.002

CI, confidence interval; logMAR: logarithm of the minimal angle of resolution; BCVA, best-corrected visual acuity.

## Discussion

In the current study, the improvement in BCVA after the initial anti-VEGF injection(s) was transient in eyes with a final Snellen BCVA of < 20/200 (the difference was not statistically significant). Advanced age, poor baseline BCVA, and comorbid internal carotid artery disease and diabetic retinopathy were risk factors for extremely poor final visual outcomes. Moreover, all patients with neovascular complications except one with vitreous hemorrhage belonged to the very poor VA group.

In the present study, approximately a third of patients exhibited a final Snellen BCVA of < 20/200; this proportion may be considerably greater than that in previous clinical trials^9–15^. Some epidemiological studies and meta-analyses showed that glaucoma^18^ and/or diabetes^19,20^ are often accompanied by CRVO. Therefore, we included CRVO patients with diabetes and/or diabetic retinopathy or glaucoma as long as they met a certain criterion; this may be a reason for the high rate of patients with very poor visual outcomes in this study. We speculate that such severe cases were excluded from the previous clinical trials^9–15^.

The number of anti-VEGF injections required in the present study appeared to be smaller than that in other clinical trials^15,17^. However, CRT showed a significant decrease from baseline in both groups (Fig. 1; Table 3). Therefore, the anti-VEGF therapies used in this study were considered effective for the suppression of retinal exudative changes to some extent. Retinal thinning due to macular ischemia could also be involved in the decrease in CRT. As previously shown, there may be a significant association between the resolution of retinal exudation and that of retinal nonperfusion^21,22^, because retinal perfusion could be the source of exudation. Inclusion of patients with severe CRVO in this study may be a reason for the smaller number of anti-VEGF injections relative to the number in other clinical trials^9–15^.

In the present study, neovascular complications, including vitreous hemorrhage, rubeosis, and NVG, occurred in 10 (6.7%), 22 (14.7%), and 16 (10.7%) eyes, respectively. These incidences are higher than those in the GALILEO and CRYSTAL studies, which included patients with not only nonischemic CRVO but also ischemic CRVO^13–15^. However, the status of the retinal circulation in patients included in the previous studies was probably not very poor, because the incidence of neovascular complications was lower than that observed during the natural course of ischemic CRVO^13^. The RAVE trial for eyes with severe CRVO also reported that VEGF inhibition by ranibizumab injections could not ameliorate the risk of such complications^17^. In a recent clinical study of eyes with BRVO, 10% of the 58 patients showed retinal or disc neovascularization during the 24-month observational period^23^. Thus, anti-VEGF treatment following a pro re nata regimen for ME, as used in the present study, may only delay the occurrence of neovascular complications.

We found that old age and comorbid internal carotid artery disease and diabetic retinopathy were significantly associated with a final BCVA of < 20/200 (Tables 2, 3, 4); these findings were partly consistent with those of Rong et al.^24^. Changes in the retinal vasculature due to age, internal carotid artery disease associated with arteriosclerosis, and diabetic microangiopathy may accelerate the deterioration of the retinal microcirculation. Retinal microcirculation has an autoregulation system that maintains a relatively constant blood flow^25^. However, if patients with internal carotid artery disease and/or diabetic microangiopathy accompanying CRVO, the autoregulation of the retinal vasculature could be easily disrupted in the retina affected by the CRVO.

This study has several limitations. First, the anti-VEGF agents and treatment protocols were not strictly unified because of the retrospective study design. Second, some of the results may not completely represent the pathology of CRVO because we included CRVO patients with systemic and other ocular disorders. Third, we could not perform OCT angiography for the assessment of macular ischemia in all cases. Therefore, the association between the macular NPA and visual outcomes was not elucidated.

Currently, anti-VEGF agents are commonly available for the treatment of CRVO associated-ME. However, in actual practice, clinicians should be aware that a certain proportion of patients with CRVO (a third in the present study) present with extremely poor final visual outcomes despite anti-VEGF treatment, as shown in the present study. Moreover, advanced age, poor baseline BCVA, and comorbid internal carotid artery disease and diabetic retinopathy are potential risk factors for extremely poor visual outcomes in patients with CRVO. Neovascular complications are not always the cause of the poor final BCVA in such cases. Future studies with larger cohorts should prospectively study the effect of anti-VEGF therapies for not only ME but also NVG in patients with CRVO.

## Methods

This retrospective study was approved by the institutional review board of Saneikai Tsukazaki Hospital (Hyogo, Japan), Kyoto University Graduate School of Medicine (Kyoto, Japan), Kagawa University Faculty of Medicine (Kagawa, Japan), and Tokushima University Faculty of Medicine (Tokushima, Japan). The procedures were in accordance with the ethical principles of the Declaration of Helsinki, and written informed consent was obtained from all included patients. We included all patients who presented at these four hospitals with ME due to acute CRVO with a symptom duration of less than 3 months before the initial treatment, between September 2013 and November 2016.

### Patients

The inclusion criteria were as follows: treatment-naïve, symptomatic, acute CRVO with retinal hemorrhage spreading over four retinal quadrants, a central retinal thickness (CRT) of > 250 µm at the initial visit, a symptom duration of < 3 months prior to examination, and availability for follow-up for over 24 months.

CRVO was diagnosed by retinal specialists at each institute on the basis of fundus examinations and fluorescein angiography (FA) findings of filing delay in the retinal veins in the four quadrants. Eyes with hemi-CRVO, BRVO, or other chorioretinal diseases, including uveitis, were excluded. Eyes that previously received any ocular treatments other than cataract surgery were also excluded. Eyes with glaucoma were included if the intraocular pressure (IOPs) was well controlled and the central visual field was intact. Patients with diabetes were included if they showed no diabetic retinopathy or mild to moderate nonproliferative diabetic retinopathy without ME.

### Study examinations

Each eye was examined every month in the first year and every 1 or 2 months in the second year at each facility. However, the interval between follow-up examinations and the follow-up duration were appropriately modified according to the disease severity.

At each visit, all patients underwent a comprehensive ophthalmic examination, including measurement of the best-corrected visual acuity (BCVA) using the Landolt chart, measurement of IOP, slit-lamp biomicroscopy, and spectral domain optical coherence tomography (OCT; Spectralis HRA + OCT, Heidelberg Engineering, Heidelberg, Germany or RS-3000, Nidek, 3D OCT-1, Topcon, Japan). Fluorescein angiography (Optos 200Tx imaging system, Optos PLC, Dunfermline, United Kingdom) was performed to assess the retinal perfusion status at the initial visit and additionally performed when deemed necessary.

### Evaluation of CRT and the defect length in the ellipsoid zone (EZ) band

For CRT measurement, a macular area in each eye was captured by spectral domain OCT. For volume OCT scanning, a whole retinal thickness map centered on the fovea was created. CRT was defined as the mean distance between the vitreoretinal interface and retinal pigment epithelium within the central subfield of the Early Treatment Diabetic Retinopathy Study grid.

To assess the integrity of the foveal photoreceptor layer, we quantified the disruption in the EZ band within the central 2-mm area on spectral domain OCT images that sectioned vertically and horizontally through the center of the fovea. The signal intensity of the EZ band was measured, and the band was quantified using the plot profile function in ImageJ software (National Institutes of Health, Bethesda, MD, USA) according to previous reports^21,26,27^. The average defect length in the EZ band was calculated from the defect lengths measured on the vertical and horizontal OCT sections.

### Intravitreal anti-VEGF injection protocols

For the treatment of ME and/or serous retinal detachment at the fovea, the patients received intravitreal anti-VEGF injections of ranibizumab (Lucentis; 0.5 mg/0.05 mL, Novartis Pharma AG, Basel, Switzerland) or aflibercept (Eylea; 2.0 mg/0.05 mL, Bayer Pharma AG; Berlin, Germany) according to different regimens in each institute. Twenty-nine eyes received one initial intravitreal anti-VEGF injection, and 121 eyes received three monthly intravitreal anti-VEGF injections. None of the patients received treatment for ME other than ranibizumab and aflibercept, such as bevacizumab injection, grid laser photocoagulation, steroid treatment, and surgical intervention.

After the initial injections in each facility, pro re nata anti-VEGF injections were administered when ME or serous retinal detachment was evident at the fovea on the OCT sections, and the patient’s consent could be obtained. The same anti-VEGF agents were used for the initial and subsequent injections, without switching of the agents. Panretinal photocoagulation (PRP) was performed when NPA extended to the posterior pole or when rubeosis and/or NVG (rubeosis with an IOP increase of > 21 mmHg) were observed.

### Medical interview and end points

For all patients, we recorded the presumed time of disease onset; age; sex; present or past history of dialysis and smoking; and present or past history of systemic diseases, including systemic hypertension, hyperlipidemia, diabetes mellitus, internal carotid artery disease, coronary artery disease, dialysis, and smoking.

According to a classification of a previous study^8^, we divided the patients into very poor VA (the final Snellen BCVA of < 20/200) and control (≥ 20/200) groups and examined the association of the final BCVA with systemic and ocular manifestations. Values determined at baseline and 1, 6, 12, and 24 months after the initial anti-VEGF injections were analyzed.

### Statistical analysis

All data were statistically analyzed using PASW Statistics, version 18.0 (SPSS, Chicago, IL, USA), and they are presented as mean ± standard deviation. For statistical analysis, VAs measured using the Landolt chart were converted to logarithm of the minimum angle of resolution (logMAR) units. Count finger, hand motion, light perception, and no light perception were assigned a logMAR value of 1.9, 2.3, 2.7, and 3.0, respectively^28^. Comparisons between the very poor VA and control groups were performed using the unpaired t-test. Comparisons between baseline BCVA and those at other time points in each group were performed using the unpaired *t*-test. The significance of systemic and ocular manifestations as risk factors for a final Snellen BCVA of < 20/200 was evaluated by calculating the adjusted ORs and 95% CIs in the multivariate logistic regression analysis, in which, the variables of diabetes and age were excluded because they were strong confounding factors. A model with lower Akaike information criterion was therefore selected. Significant differences in the sampling distributions were determined using chi-square tests. A *P* value of < 0.05 was considered statistically significant.

## Data Availability

The datasets generated during and/or analyzed during the current study are available from the corresponding author on reasonable request.
